# Machine Learning: How Much Does It Tell about Protein Folding Rates?

**DOI:** 10.1371/journal.pone.0143166

**Published:** 2015-11-25

**Authors:** Marc Corrales, Pol Cuscó, Dinara R. Usmanova, Heng-Chang Chen, Natalya S. Bogatyreva, Guillaume J. Filion, Dmitry N. Ivankov

**Affiliations:** 1 Genome Architecture, Gene Regulation, Stem Cells and Cancer Programme, Centre for Genomic Regulation (CRG), Barcelona, Spain; 2 Universitat Pompeu Fabra (UPF), Barcelona, Spain; 3 Spain Genome Architecture, Gene Regulation, Stem Cells and Cancer Programme, Centre for Genomic Regulation (CRG), Barcelona, Spain; 4 Bioinformatics and Genomics Programme, Centre for Genomic Regulation (CRG), Barcelona, Spain; 5 Moscow Institute of Physics and Technology, Dolgoprudny, Moscow Region, Russia; 6 Laboratory of Protein Physics, Institute of Protein Research of the Russian Academy of Sciences, Pushchino, Moscow Region, Russia; University of Leeds, UNITED KINGDOM

## Abstract

The prediction of protein folding rates is a necessary step towards understanding the principles of protein folding. Due to the increasing amount of experimental data, numerous protein folding models and predictors of protein folding rates have been developed in the last decade. The problem has also attracted the attention of scientists from computational fields, which led to the publication of several machine learning-based models to predict the rate of protein folding. Some of them claim to predict the logarithm of protein folding rate with an accuracy greater than 90%. However, there are reasons to believe that such claims are exaggerated due to large fluctuations and overfitting of the estimates. When we confronted three selected published models with new data, we found a much lower predictive power than reported in the original publications. Overly optimistic predictive powers appear from violations of the basic principles of machine-learning. We highlight common misconceptions in the studies claiming excessive predictive power and propose to use learning curves as a safeguard against those mistakes. As an example, we show that the current amount of experimental data is insufficient to build a linear predictor of logarithms of folding rates based on protein amino acid composition.

## Introduction

Understanding the self-organization of protein structure is one of the most important problems of the last 50 years in biophysics [1]. Massive experimental and theoretical efforts have led to a better understanding of protein folding [2] culminating in successful predictions of protein structures [3–6] and *de novo* protein design [7]. In the light of this spectacular progress, apparently simpler tasks still remain problematic. One of them is predicting the rate of protein folding, *i*.*e*. the speed at which a protein renatures *in vitro* in conditions matching the physiology. Surprisingly, proteins fold fast (from microseconds [8] to hours [9]) even though the number of conformations is astronomical. This fact, known as the Levinthal paradox [10], remained unexplained until the discovery of nucleation mechanism [11]. Nucleation-based model solved the paradox, while predicting that the time required to fold a protein is proportional to *L* ^*2/3*^, where *L* is the number of residues [12,13]. In contrast, the influence of the protein topology on the folding was discovered empirically [14]. Developing methods to predict protein folding rate may highlight unknown determinants of protein folding and lead to a detailed understanding of how proteins self-organize.

Predictive methods usually provide an estimate for the natural logarithm of the folding rate of a protein (here referred to as the log folding rate), and they are typically scored using the correlation between the predicted log folding rate and the actual log folding rate. For convenience, we will refer to this score as the “correlation of the model”, even though it is a joint property of the model and of the training set. It has long been observed that the folding rate of a protein strongly depends on its length [13,15,16]. Consequently, models that predict the log folding rate using only the length of a protein can reach correlations as high as 0.70 [17]. By using the topology of the protein [14], the correlation can be further improved [18]. Can more sophisticated approaches make better predictions, and if so, what key features must be taken into account?

The ongoing accumulation of experimental data [14,19–22] has accelerated the development of statistical and machine learning methods to address this question [23–34]. Those studies claim correlations ranging from 0.74 [27] to 0.99 [24], among which many lie above 0.90 [24,29–33]{FormattingCitation}. Here, we tested three of those models [24,26,29] against recently collected experimental data. We found much lower predictive powers than the original claims. In all instances, the unifying cause was overfitting, an umbrella term describing situations where models perform well on training data and poorly on new data. Based on this, we suggest that claims of high correlations should be taken with caution and that future studies should demonstrate that they do not suffer from overfitting by using learning curves.

## Results

### Data set

We collected folding rates obtained experimentally using two references [22,35]. The whole data set contains 113 single-domain proteins without disulphide bonds; 74 of those have two-state folding kinetics in physiological conditions, and the remaining 39 have multi-state kinetics (S1 Table). Here on we refer to this combined data set as “data set 113”.

### Small sample singularities

**Fig 1:**
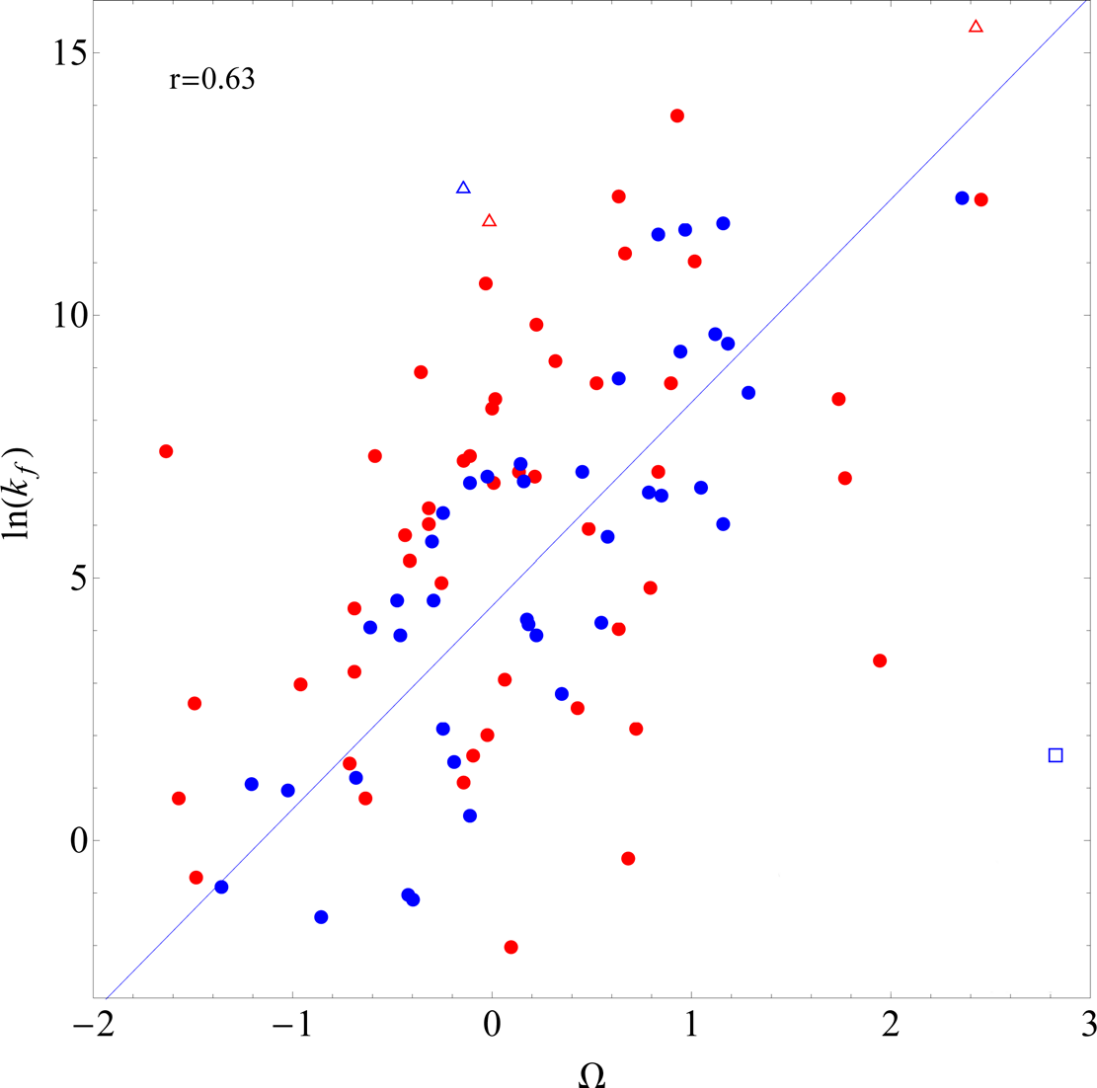
Correlation of Huang and Tian’s model. The correlation between Ω, the sum of amino acid foldabilities proposed in [26], and the log folding rates for two-state proteins. Blue dots represent proteins from the data set of Huang and Tian [26]. Red symbols show two-state proteins from data set 113. Correlation coefficients were calculated using only proteins of length comprised between 30 and 200 residues, depicted as circles (0.82 for Huang and Tian’s set and 0.63 for two-state proteins from data set 113). Proteins with fewer than 30 amino acid residues are shown as triangles, while those with more than 200 residues are shown as squares. The line shows the prediction from the original model by Huang and Tian [26].

In a study by Huang and Tian [26], the authors introduce a parameter Ω for each amino acid, defined as the sum of its rigidity R [36] and its dislike for all regular secondary structures D. D is calculated as a linear combination of parameters P_α_, P_β_ and P_turn_ [37], which measure the resistance of each amino acid type to form α-helix, β-sheet and reverse turn, respectively. The authors estimated the three parameters by fitting a linear regression model on experimental log folding rates. Summing Ω over all the amino acids of the protein, they obtain a total Ω, used as a predictor of the log folding rate. The reported correlation of the model for 40 two-state proteins from 30 to 200 residues long is equal to 0.82 (blue circles in the Fig 1).

We benchmarked the model of Huang and Tian using log folding rates of proteins from data set 113 (red circles in the Fig 1). On this new data set, the model achieved a correlation of 0.63. Here we took care of including only two-state proteins, as the model makes claims for this category only. For the same reason, we included proteins of length comprised between 30 and 200 residues only. Thus, the discrepancy is not due to extrapolation beyond the domain of validity of the model.

To understand the difference with the original claim, it is important to realize that linear regression is an estimation problem. In other words, the coefficient of correlation is a random variable with an inherent uncertainty due to sampling. A widespread misconception is that unbiased estimators are exact. The fundamental issue here is that the estimators are inaccurate due to the low amount of experimental data in the training set. The correlation can be high because the training set accidently contains an unusually high number of proteins that strictly follow the model of Huang and Tian.

**Fig 2:**
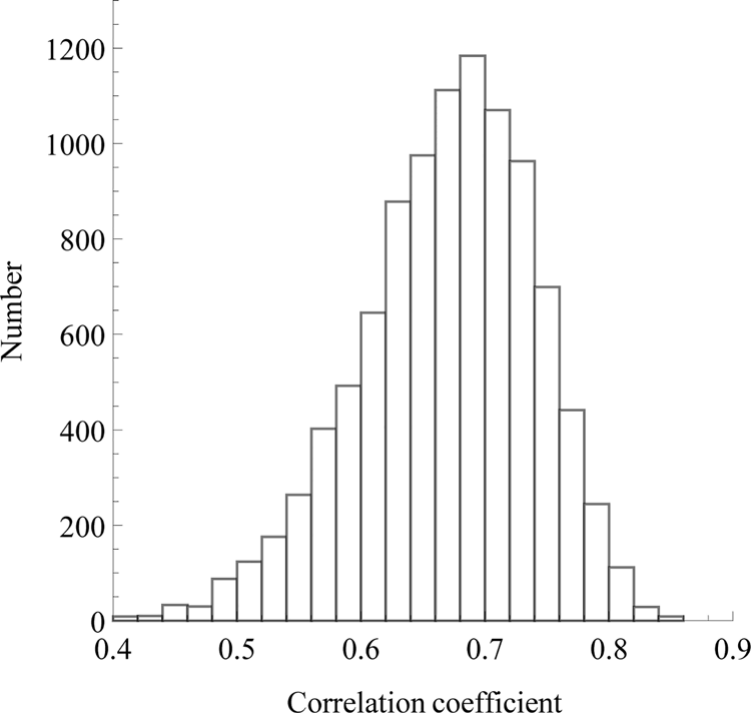
Correlation coefficient of Huang and Tian’s model for different samples. Forty data points were randomly sampled from a meta data set and the model described by Huang and Tian [26] was fitted again 10,000 times. The meta data set consists of two-state proteins from 30 to 200 residues combined from [26] and data set 113, without duplicates. The histogram of the obtained correlation coefficients was then plotted. The correlation coefficient ranges from 0.5 to 0.8 approximately, which shows that robust estimation of the correlation cannot be achieved with 40 proteins.

To show this, we combined the original training data set from [26] with data set 113, removed duplicates, and sampled 40 proteins at random (the number of proteins of the original training set) in order to fit again the model of Huang and Tian. As can be seen on Fig 2, the coefficients of correlation thus obtained fluctuate widely; most of the values are comprised in the range 0.5–0.8.

A danger of statistical approaches is that the training sample may not be representative of future data sets. Modelling peculiarities found only in the training set will result in overfitting. With a correlation equal to 0.82, the training set used by Huang and Tian is an outlier (*p* = 0.004), explaining why the predictive power was low on new data. In general, smaller samples have more chances of being aberrant. With 40 data points, the authors may have found a correlation anywhere between 0.5 and 0.8. Unfortunately, one cannot know that a sample used for training is non-representative before acquiring new data. But one can discover that this is *possible* by inspecting the variance of the estimates. Learning curves can be used for this purpose, as we suggest below.

### Overtraining

In another study by Gromiha, the author predicts the log folding rates of proteins from physical and conformational properties of their amino-acids [24]. Separating proteins based on their secondary structures (“alpha-helices only”, “beta-sheets only” or mixed) he obtains three models, with a range of correlations between 0.95 and 0.99. In a later study by Gromiha, Thangakani and Selvaraj, the authors use the same approach on a larger data set and obtain correlations between 0.90 and 0.99 [29].

We challenged the models of the first study with the proteins of data set 113 and obtained a predictive power much lower than claimed [24]. The coefficients of correlation for categories “alpha-helices only”, “beta-sheets only” and mixed were -0.28, 0.008 and 0.02, respectively. The first measure is negative, but not significantly different from 0 (correlation test, p = 0.147), so it should be interpreted as no evidence for statistical association. The last two numbers speak for themselves.

The parameters of the second study are not given explicitly but the authors provide the model as web service [29]. The interface conveniently allows to submit inputs of unknown structural class. Using this option, we submitted the proteins of data set 113 and obtained a correlation coefficient with the measured log folding rates equal to 0.14. In summary, those models have no or very modest predictive power. How to explain that they had spectacular performance on the earlier data sets?

The common issue between those models is overtraining. When the same training set is used many times, the risk is that a model accidentally captures the singularities of the data set. This risk increases when the training set is small and when the number of trainings is large. In the first study for instance, the training set for “beta-sheets only” consisted of 13 proteins [24], and it was used to train over 2 million models (each with 3 to 6 parameters).

Overtraining is a ubiquitous risk in machine learning because it is easy to perform unwittingly. The standard approach to reducing this risk is to separate the data into a training set and a testing set. The training set is used to choose a predictive model, and the testing set is used to evaluate its predictive power. In the variant of this approach called cross-validation, the procedure is repeated and averaged, so that the same data point may be used for training and for testing. Cross-validation is a good statistical practice but not a guarantee, as even cross-validated models may be overtrained.

**Fig 3:**
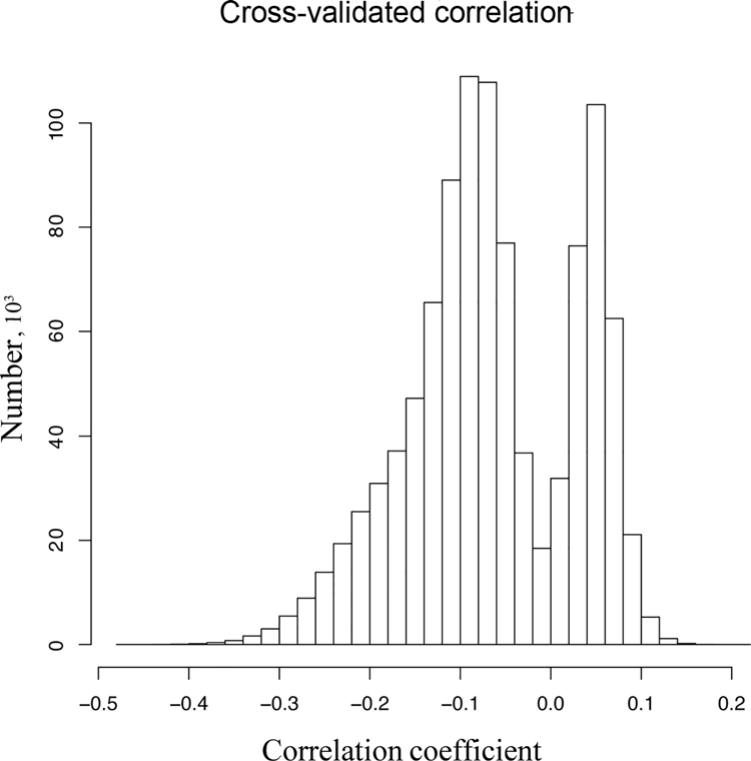
Cross-validation results for two independent Gaussian samples. In this toy model, we try predict a variable from an uncorrelated predictor. The predictive power is null, but the model can be overtrained and give the illusion that the variables are correlated. We repeatedly performed 5-fold cross validation 1,000,000 times on the same data set (n = 100). The plot shows the distribution of the obtained coefficient of correlation. The highest value is 0.202, and the lowest is -0.472 (associated *p*-values without multiple-hypothesis correction equal to 0.044 and 7·10^−7^, respectively).

To illustrate this point, we generated two Gaussian samples with a pseudo-random generator. Being generated separately, the samples are independent. Consistently, the measured correlation between them was -0.0495 (correlation test, *p* = 0.625). When then performed 5-fold cross-validation as in [34] by segmenting the samples in five blocks, using four to train a linear model and measuring the predictive power on the fifth. There are many ways to segment the sample in 5 blocks, and there is as much flexibility to choose a partition where the predictive power is abnormally high. In our example, 1,000,000 tested partitions gave the best correlations equal to 0.20 (positive) and -0.47 (negative), associated *p*-values without multiple-hypothesis correction equal to 0.044 and 7·10^−7^, respectively (Fig 3). Claiming that these models have cross-validated correlation equal to 0.20 and -0.47 is true but harmful, since there is in reality no statistical association between the variables. As in other cases of overfitting, the definitive criterion is to measure the predictive power on new data, that is to say data that were never “seen” by the model at any stage of its construction.

### How much can be achieved?

How much predictive power can be achieved from statistical and machine learning-based methods is an open question. In particular, it is often impossible to establish a hard limit between the achievable and the non-achievable. In this section, we focus on composition-based predictions of the log folding rate because the number of features is small enough for an exhaustive linear fit. We consider the most complete linear model based on amino-acid composition, that is to say, the one that consists of one parameter per amino-acid. In this model, each amino-acid type brings its own contribution to the log folding rate of the protein. This model contains 21 parameters and extends *every* linear model based on amino-acid composition.

**Fig 4:**
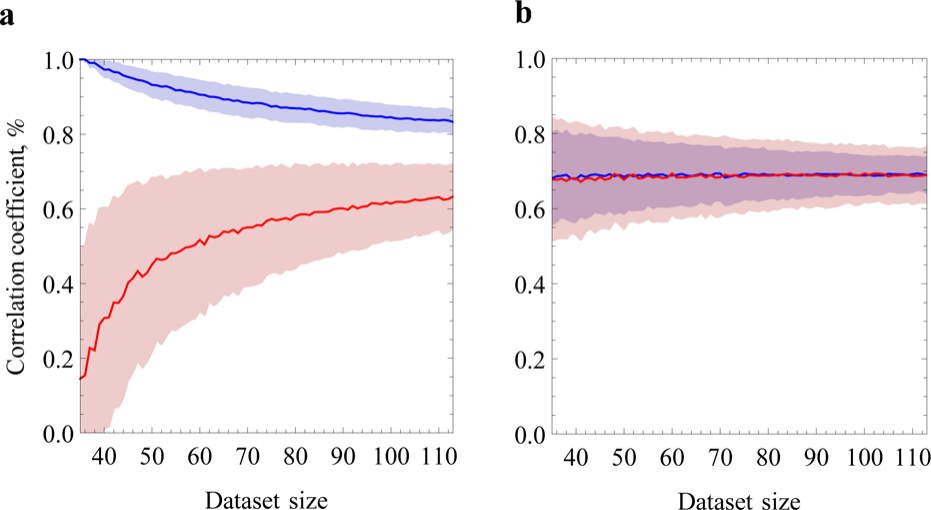
Learning curves of the linear regression model. The mean (n = 1000) correlation coefficient of the training and test sets between the predicted and observed log folding rates (blue and red lines, respectively) is plotted as a function of the dataset size, together with the standard deviations of both sets (blue and red regions, respectively). Sixty percent of the examples are assigned to the training set and 40% to the test set. **a.** Log folding rates were fitted with 20 features corresponding to the absolute amino acid frequency of each protein. A clear overfit can be seen as a gap between the two correlation lines. **b.** Log folding rates were fitted using a single feature corresponding to the amino acid length of each protein to the power of 2/3, ln(*k*_*f*_) ~ -*L* ^2/3^ [13]. There exists a nearly-perfect correspondence between training and test sets, and a slightly higher correlation on the test set than in Fig 4A.

We fitted this model with an increasing number of data points and plotted the learning curves in Fig 4A. The learning curve consists of the amount of explained variance R^2^ (the square of the coefficient of correlation) on a test data set and on the data set used for training. As the sample size increases, there are more points to fit in the training set with the same number of parameters, so the explained variance decreases (blue). Meanwhile, the model becomes more general and acquires more predictive power on new data, so the explained variance on the testing set increases (red). The vertical distance between the two lines shows the extent of overfitting, or in other words the lack of fit of the model when confronted with new data. When the two curves meet, the model is not overfitted and the true predictive power is the value of the common asymptote. Note that for consistency with the previous sections, we plotted the correlation coefficient instead of the more common R^2^. The two lines do not converge, even when all the data points available are included, which means that there is presently not enough experimental data to properly train the complete model.

On the other hand, we used one of the simplest existing model to fit the same data, namely the nucleation-based model ln(*k*_*f*_) ~ -*L* ^2/3^ [13], which only takes into account the size of the protein *L*. For this model, the learning curve shows that the explained variances on the training and test sets are indistinguishable (Fig 4B), which means that the model is not overfitted (for *L* ^1/2^ [16] and ln(*L*) [15] the curves are indistinguishable as well, data not shown). However, the performance corresponds to a correlation around 0.70 with a significant uncertainty around this value even when all the data points are included.

**Fig 5:**
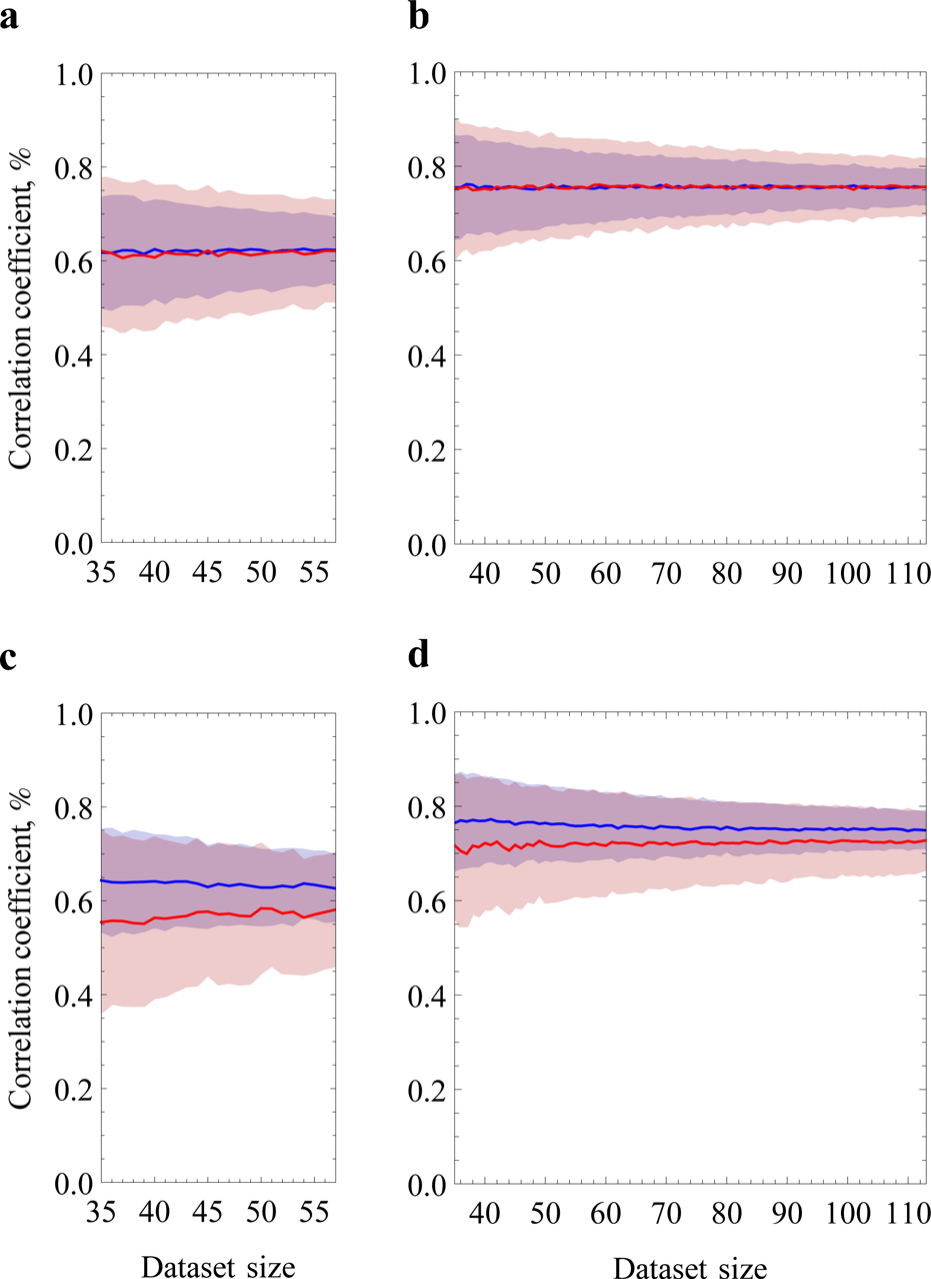
Learning curves of the contact order models. **a**. Relative contact order model with fixed parameters *d* and *ΔL* (atoms contact in three-dimensional protein structure if they are closer than *d* = 6Å and belong to the residues having distance by chain *ΔL* ≥ 1). **b**. Absolute contact order model with fixed parameters *d* and *ΔL*. Relative (**c**) and absolute (**d**) contact order models with varying parameters *d* and *ΔL*. For relative contact order model we restrict the data set to two-state proteins having less than 150 residues.

Using the same learning curve approach, we tested slightly more complicated models based on contact order [14,18,38]. Absolute contact order is the average number of residues separating by chain the atoms contacting in protein structure [18,38]. Relative contact order is a further normalization of absolute contact order by the number of residues, thus describing the average fraction of protein residues separating the atoms contacting in the structure [14]. For the purpose of calculating contact order, atoms are assumed to be in contact if they are closer than *d* = 6Å and the chaining distance between corresponding residues *ΔL* ≥ 1, which is reasonable from the physical point of view. With fixed parameters *d* and *ΔL*, the learning curves are indistinguishable, as with *L* ^2/3^ (Fig 5A and 5B). If parameters *d* and *ΔL* are allowed to vary, the learning curves diverge slightly (Fig 5C and 5D). This slight overfit means that even for a model with three parameters, the currently available amount of experimental data can be an issue.

In summary, the lack of experimental data on the rate of protein folding is such that model fitting suffers large fluctuations, even for models with few parameters.

## Discussion

The abstraction of protein folding rate has reduced the complex process of protein folding to a single number, making it easy to formalize for machine learning tasks. The regular releases of experimental folding rates [14,19–22,35] makes the prediction of protein folding rates a tempting task, especially with the use of machine learning techniques, where many models can be proposed regardless of their interpretation. The additional possibility to split proteins into different structural classes or into two- and multi-state proteins (which proved initially useful in understanding protein folding principles [14,18]) makes the task even easier. While these tasks are easy to perform, it is equally easy to make a mistake while performing them.

When challenging published models with new data, we discovered that the claims to predict the log folding rate with a correlation higher than 0.90 were too optimistic because the models were overfitted. More precisely, smallish data set and overtraining were the major sources of overfitting. It is worth mentioning that we did not find any example of overparameterization, which is another well-known pitfall of machine learning. With 113 experimental folding rates, the data set used in this study is one of the largest available. This is far from “big data”. Methods of prediction and feature extraction that have proved successful for larger data sets may not be directly applied to folding rates. Or, more correctly, not until more data is available. In the current context, hypothesis-driven approaches are more called for.

The established determinants of protein folding are protein size and topology [13–16,18]. We argue that the low amount of experimental data currently prohibits discovering more subtle determinants of protein folding rates by statistical and machine-learning methods. The scarcity of experimental data makes it easier to be the victim of overfitting. As a recommendation for future studies, we suggest to use learning curves to demonstrate the validity of the models instead of correlations and *p*-values.

## Methods

### Data set

We collected folding rates obtained experimentally using two references [22,35]. The whole data set contains 113 single-domain proteins without disulphide bonds (“data set 113”). Seventy four of those have two-state folding kinetics at physiological conditions, and the remaining 39 have multi-state kinetics (S1 Table). In order to reproduce the method developed in [26], we took amino acid compositions, sum of Ω´s and log protein folding rates from the Supplementary Table 2 of ref. [26] for 42 records used therein. Huang and Tian excluded two proteins from the final fit, thus leaving 40 proteins for analysis.

### Analyses

For one-parameter linear regression fit we used “foldability” Ω. Huang and Tian introduced this parameter as a sum of amino acid rigidity and its dislike for all regular secondary structures [26]. They also determined Ω for each amino acid type. Summing Ω values of all residues of protein we calculated the total Ω. FOLD-RATE [29] was queried by a custom Bash script performing an HTTP POST request containing the sequence of the protein of interest and parsing the html response from the server. Briefly, wget was run with options http://psfs.cbrc.jp/cgi-bin/fold-rate/foldrateCalculator.pl—postdata = "sequence = $seq&eqn = unknown". All the linear regression analyses were performed using the lm() function in R with default parameters. Correlations were likewise computed with the cor() function of R.

To give a lower bound on the number of trainings from [24], we used the following passage from the text “As the single property with the highest r-value is not sufficient for accurate prediction I have combined different amino acid properties with a multiple regression fit. The computation has been carried out with the combinations of two to five amino acid properties”. There are 2,138,360 different ways to choose two to five amino acid properties among 49, representing as many different models, with 3 to 6 parameters each.

## Supporting Information

S1 Table The list of proteins used in the paper. (PDF)
