# Supplementary material for: Machine Learning: How Much Does It Tell about Protein Folding Rates?
Source: PLoS One. 2015 Nov 25;10(11):e0143166. doi: 10.1371/journal.pone.0143166 (PMC4659572; doi:10.1371/journal.pone.0143166)
Supplement: S1 Table — (PDF) [file pone.0143166.s001.pdf]

Supplementary Table 1. The list of proteins used in the paper.

	name	PDB	length	ln(k_f)	kinetic order	Ω	amino acid composition																			
							A	C	D	E	F	G	H	I	K	L	M	N	P	Q	R	S	T	V	W	Y
1	Trp-cage protein	1L2Y	20	12.5	two	-0.14	0	0	1	0	0	3	0	1	1	2	0	1	4	1	1	3	0	0	1	1
2	Alanine-based peptide	alpha	21	15.5	two	2.43	18	0	0	0	0	0	0	0	0	0	0	0	0	0	3	0	0	0	0	0
3	Villin headpeace subdomain	1VII	36	9.4	two	0.83	3	0	2	2	4	2	0	0	5	5	2	2	1	2	1	2	1	1	1	0
4	Peripheral subunit-binding domain	2PDD	41	9.8	two	0.23	5	0	3	2	1	6	0	2	4	3	1	1	1	1	4	1	1	4	0	1
5	GA module of albumin binding domain	1PRB	47	13.8	two	0.93	9	0	2	5	2	1	1	5	6	4	0	5	0	0	0	1	2	3	0	1
6	B-domain of staphylococcal proteinA	1BDD	58	11.7	two	1.65	7	0	4	5	3	1	1	2	6	7	0	8	3	6	1	3	0	0	0	1
7	Engrailed homeodomain	1ENH	61	10.5	multi	0.95	4	0	0	7	3	2	0	3	7	6	0	4	1	5	9	5	3	0	1	1
8	TRF1 Myb domain	1BA5	53	5.9	two	0.49	1	0	2	3	1	3	1	1	8	6	2	4	0	1	7	3	2	2	4	2
9	RAP1 Myb domain	1FEX	59	8.2	two	0.00	6	0	4	2	1	3	2	2	5	6	1	2	1	2	4	7	4	3	2	2
10	c-Myb-transforming protein	1GV2	55	8.7	two	0.90	4	0	2	7	0	3	2	4	4	4	1	4	1	3	6	1	3	1	3	2
11	Phage 434 cro protein	2CRO	71	3.7	multi	1.40	8	1	1	4	2	4	0	4	8	8	3	1	2	6	6	2	6	3	1	1
12	α3D	2A3D	73	12.2	two	2.46	15	0	1	11	3	5	1	3	6	7	1	2	1	4	5	3	1	1	1	2
13	l-repressor	1LMB	81	10.4	two	1.48	10	0	2	9	2	6	0	5	7	10	2	5	2	4	3	6	1	4	1	2
14	Acyl-coenzyme A binding protein	1NTI	86	7.0	two	0.84	9	0	7	10	3	5	2	4	15	5	3	2	2	2	1	3	4	3	2	4
15	Hypothetical protein encoded by the Yjbj gene from E.coli	1JYG	89	9.1	two	0.32	2	1	7	8	1	9	7	3	10	3	3	3	1	6	5	5	3	5	4	3

16	Colicin E9 immunity protein	1IMQ	93	7.3	two	-0.11	4	1	6	11	3	6	9	4	6	6	2	3	3	3	1	9	7	5	1	3
17	Colicin E7 immunity protein	1AYI	94	7.2	two	-0.14	5	0	9	12	3	5	8	6	8	7	1	4	4	2	2	4	4	6	1	3
18	Cytochrome b562	256B	106	12.3	two	2.24	17	0	12	8	2	3	2	3	13	10	3	6	4	6	4	2	5	4	0	2
19	15th domain of brain α-spectrin	1U5P	118	11.0	two	1.02	13	0	12	10	5	3	2	2	13	15	1	6	0	8	3	14	3	5	2	1
20	16th domain of brain α-spectrin	1U5P	118	4.8	two	0.80	9	0	10	11	3	7	4	6	13	11	2	4	1	7	11	8	5	4	1	1
21	17th domain of brain α-spectrin	1CUN	118	3.4	two	1.95	12	1	7	16	4	6	5	5	11	10	2	7	0	8	4	3	5	8	2	2
22	Tumour suppressor protein p16	2A5E	148	3.5	multi	3.65	27	1	11	10	1	13	5	3	0	17	4	4	10	1	17	5	5	10	2	2
23	Apomyoglobin	1A6N	151	1.1	multi	1.10	17	0	7	14	6	10	12	9	19	18	2	1	4	4	4	6	5	8	2	3
24	Lyme desease variable surface antigen	1L8W	338	2.0	two	2.57	60	0	23	26	10	42	0	15	43	17	2	12	8	6	3	27	17	25	0	2
25	C-terminal β-hairpin of protein GB1	1PGB	16	12.0	two	-0.42	1	0	2	2	1	1	0	0	1	0	0	0	0	0	0	0	5	1	1	1
26	Pin WW domain	1PIN	34	9.5	two	0.15	1	0	0	2	1	3	1	1	2	1	1	2	3	1	4	5	1	1	2	2
27	Prototype WW domain	1E0M	38	8.9	two	-0.35	0	0	2	1	0	4	2	0	3	1	2	3	3	0	1	4	6	0	2	4
28	Formin-binding protein	1E0L	37	10.6	two	-0.03	4	0	1	5	0	2	0	0	4	2	0	2	1	1	1	2	6	1	1	4
29	Yes kinase-associated protein	1JMQ	40	8.4	two	0.02	2	0	4	2	2	2	1	2	2	2	1	1	4	3	2	2	4	1	2	1
30	Src SH3 domain	1RLQ	62	4.4	two	-0.69	4	0	4	4	2	5	1	2	2	6	0	3	2	2	2	6	8	3	2	4
31	α-spectrin SH3 domain	1SHG	62	1.1	two	-0.14	3	0	6	5	1	3	0	1	8	7	2	3	2	2	2	2	4	6	2	3
32	Cold shock protein B	1C9O	66	7.2	two	0.14	3	0	1	8	6	10	1	3	5	2	1	4	1	5	2	2	2	8	1	1
33	Cold shock protein	1G6P	66	6.3	two	-0.25	3	0	3	8	5	9	2	3	10	1	2	0	1	3	1	2	2	8	2	1

	B																									
34	Cold shock protein B	1CSP	67	6.5	two	0.45	5	0	2	10	7	9	1	3	5	2	1	3	1	4	1	3	2	7	1	0
35	Actin binding protein ABP1 SH3 domain	1JO8	68	2.5	two	0.43	5	0	8	8	3	4	6	3	3	6	1	5	2	0	0	3	2	3	3	3
36	Photosystem I accessory protein E	1PSF	69	3.2	two	-0.69	4	0	2	5	3	9	1	6	5	3	0	6	1	0	3	6	2	8	1	4
37	Cold shock protein A	1MJC	70	5.3	two	-0.41	5	0	6	2	6	10	1	4	7	2	2	3	2	2	0	7	4	5	1	1
38	Fyn SH3 domain	1AVZ	78	4.9	two	-0.25	4	0	4	7	3	7	8	2	2	6	2	2	3	1	3	10	5	3	2	4
39	SH3-like domain of virulence protein internalin B	1M9S	85	4.0	two	0.64	5	1	3	6	0	6	7	3	8	10	1	6	1	1	8	3	7	3	2	4
40	Immunoglobulin domain of cardiac titin	1TIT	89	3.6	multi	1.05	7	2	3	11	3	7	4	6	8	10	1	3	4	4	0	4	3	7	1	1
41	Ninth fibronectin type III module of fibronectin	1FNF	90	-0.9	two	-1.00	4	0	4	5	3	7	5	8	0	6	0	4	7	2	8	9	10	5	1	2
42	Third fibronectin type III repeat of tenascin	1TEN	92	1.1	two	-1.26	4	0	10	8	2	6	0	8	5	7	1	3	5	2	4	7	12	4	1	3
43	SH3 domain of the p85α subunit of phosphatidylinositol 3'-kinase	1PNJ	90	-1.0	two	-0.40	4	0	6	10	2	12	1	5	6	7	1	4	3	2	4	7	4	3	2	7
44	18th module of muscle protein twitchin	1WIT	93	0.4	two	-0.12	11	0	6	6	5	6	1	7	10	8	0	3	5	1	2	7	7	6	1	1
45	Tenth fibronectin type III domain of	1FNF	94	5.5	multi	-2.05	7	0	5	4	1	8	0	7	3	4	0	2	8	1	5	10	12	10	1	6

	fibronectin																									
46	C-terminal domain of the cell-surface receptor protein CD2	1CDC	98	1.8	multi	-0.28	5	0	9	4	4	10	1	6	7	10	2	7	2	1	7	6	8	5	2	2
47	Ileal lipid binding protein	1EAL	127	1.3	multi	-0.09	4	1	6	14	8	13	1	7	13	3	5	6	2	5	5	8	9	11	1	5
48	Intestinal fatty acid binding protein	1IFC	131	3.4	multi	-0.01	6	0	7	14	8	12	1	7	15	9	3	10	0	2	6	4	10	11	2	4
49	Cellular retinol binding protein II	1OPA	134	1.4	multi	0.19	3	3	11	11	7	11	1	4	15	9	2	9	0	7	6	2	14	11	4	3
50	Cellular retinoic acid binding protein I	1CBI	137	-3.2	multi	1.21	9	3	8	14	6	9	2	6	9	10	3	7	4	3	9	5	14	10	3	3
51	Chemotaxis protein CheW	1K0S	151	7.4	two	-1.63	6	0	13	13	7	9	1	18	15	15	3	5	3	3	7	7	7	18	0	1
52	Cyclophilin A	1LOP	164	6.6	two	0.97	10	2	11	11	12	16	5	10	11	5	6	12	5	6	5	6	12	15	1	3
53	Barstar	1BRS	89	3.4	multi	0.43	8	0	4	11	2	5	1	6	6	12	0	3	2	6	3	5	4	5	3	3
54	Chemotactic protein	1DJM	129	1.0	multi	2.43	16	0	8	11	6	10	0	6	11	15	7	8	3	2	4	4	5	10	1	2
55	C-terminally truncated fragment of GroEL apical domain	1AON	155	-1.5	multi	1.01	17	0	8	16	4	15	0	14	11	15	5	4	6	4	7	4	10	13	0	2
56	Ribonuclease HI	1F21	155	0.1	multi	1.80	17	0	7	12	2	14	5	7	11	12	4	7	5	8	10	4	10	9	6	5
57	Dihydrofolate reductase	4KJK	159	-3.2	multi	1.50	13	2	13	12	6	10	5	12	6	11	5	6	10	4	9	9	6	11	5	4
58	N-terminal domain of phosphoglycerate kinase	1PHP	174	2.3	multi	2.37	23	1	12	17	6	12	5	7	12	18	3	7	7	1	14	2	6	17	0	4
59	C-terminal domain of phosphoglycerate	1PHP	220	-3.5	multi	0.78	26	0	19	16	9	23	3	14	20	20	6	6	8	1	6	9	7	21	1	5

	kinase																									
60	Tryptophan synthase α subunit	1QOP	268	-2.5	multi	4.65	40	3	13	17	12	19	4	20	13	27	5	9	19	12	11	11	9	17	0	7
61	Tryptophan synthase β2 subunit	2DH5	397	-6.9	multi	5.95	41	5	17	30	13	43	14	23	18	39	15	14	18	16	18	18	23	19	1	12
62	BBA5 mini-protein	1T8J	23	11.8	two	-0.01	2	0	2	1	0	1	1	0	1	3	0	0	1	1	3	3	0	0	1	3
63	N-terminal domain from ribosomal protein L9	1DIV	56	6.6	two	1.03	7	0	2	4	3	5	0	3	11	5	1	4	1	4	1	0	1	3	0	1
64	B1 domain of streptococcal protein G	3GB1	62	6.3	two	-0.31	6	0	5	5	2	4	6	1	6	3	1	3	0	1	0	0	11	4	1	3
65	Sso7d protein, Y34W	1BNZ	64	6.95	two	0.01	3	0	3	7	2	7	0	3	14	3	3	0	1	2	2	3	3	5	2	1
66	Chymotrypsin inhibitor 2	2CI2	65	5.8	two	-0.43	3	0	4	6	1	3	0	6	6	6	2	1	4	3	4	1	3	10	1	1
67	Ubiquitin	1UBQ	76	7.3	two	-0.58	2	0	5	6	2	6	1	7	7	9	1	2	3	6	4	3	7	4	0	1
68	Immunoglobulin light chain-binding domain of protein L	1HZ5	72	4.1	two	0.72	10	0	4	6	4	5	6	3	7	3	2	4	0	1	0	2	8	3	1	3
69	Ras-binding domain of C-raf-1	1RFA	80	8.4	two	1.74	5	3	5	4	3	5	3	2	6	11	3	5	2	3	6	3	3	7	1	0
70	Activation domain of procarboxypeptidase A2	1O6X	81	6.8	two	0.01	4	0	3	9	4	3	2	5	3	8	2	3	4	8	2	5	4	10	1	1
71	Histidine-containing phosphocarrier protein	1POH	85	2.7	two	0.34	9	0	1	9	4	6	2	3	7	8	2	2	2	6	1	6	10	7	0	0
72	N-terminal domain of HypF	1GXT	91	4.4	multi	0.91	3	3	5	6	5	7	2	4	2	9	1	3	6	8	8	3	4	9	2	1

73	C-terminal domain from ribosomal protein L9	1DIV	92	3.3	two	0.63	12	0	2	9	1	6	3	5	12	12	0	2	3	5	3	3	7	6	0	1
74	Ribosomal protein L23	1N88	96	2.0	two	-0.02	11	0	2	6	3	7	2	5	16	7	1	2	4	2	5	1	6	11	1	4
75	Common-type acylphosphatase	2VH7	98	0.8	two	-0.63	3	0	5	6	5	10	3	6	10	7	1	5	2	8	4	5	5	9	2	2
76	Muscle acylphosphatase	1APS	99	-1.6	two	-1.22	2	1	4	6	4	9	0	4	9	3	2	5	2	4	6	14	7	11	2	4
77	Ribosomal protein S6	1RIS	101	6.1	two	1.48	8	0	5	10	2	4	0	5	4	12	3	7	5	6	12	2	0	10	1	5
78	Spliceosomal protein U1A	1AUD	102	4.6	two	0.58	7	0	5	4	6	4	2	10	11	7	5	5	4	4	6	8	5	5	1	3
79	Barnase	1BNI	110	2.6	multi	-0.95	8	0	9	3	4	10	2	8	8	7	0	6	3	4	6	9	9	4	3	7
80	FK506 binding protein	1FKF	109	1.6	two	-0.09	5	1	6	6	5	15	3	5	8	7	4	1	7	5	6	6	7	9	1	2
81	Src SH2 domain	1IS0	110	8.7	two	0.53	6	3	4	7	5	8	3	3	8	11	0	5	4	5	8	10	8	5	1	6
82	Cell-cycle regulatory protein p13suc1	1PUC	113	4.2	multi	0.67	4	0	5	13	4	7	4	6	6	11	5	1	8	6	9	6	5	4	2	7
84	T4 lysozyme	212L	164	4.1	multi	1.65	17	0	10	8	5	10	1	11	13	16	5	12	3	5	13	6	11	9	3	6
85	Sho1 SH3 domain	2VKN	76	2.1	two	0.73	7	0	7	8	1	4	7	7	3	4	2	3	3	3	4	3	1	2	2	5
86	Ubiquitin related modifyer 1	2PKO	101	2.6	two	-1.49	1	0	13	7	4	10	3	14	6	7	3	5	3	1	2	6	6	8	1	1
87	Hypothetical protein Tm1083	1J5U	124	6.9	two	0.22	6	0	8	21	5	6	2	10	13	12	2	4	3	0	7	3	9	6	3	4
88	Villin 14T, N-terminal domain of villin	2VIK	126	4.2	multi	-0.42	6	1	4	9	4	14	3	6	10	7	4	4	3	8	4	10	9	9	2	9
89	E3-binding domain of BBL	2WXC	47	11.2	two	0.67	7	0	3	3	0	5	1	2	3	6	0	3	1	1	4	3	2	2	1	0
90	LysM domain	1E0G	66	7.0	two	0.14	4	0	6	0	1	4	1	3	5	5	2	8	2	2	5	9	3	4	1	1

91	POB	1W4J	51	12.3	two	0.64	10	0	2	5	0	6	0	2	5	2	1	0	2	0	4	2	4	5	1	0
92	Pit1 homeodomain	1AU7	63	9.7	multi	1.37	4	1	1	9	2	1	2	3	7	3	2	3	1	2	11	4	2	4	1	0
93	FF domain	1UZC	71	7.7	multi	1.34	9	0	1	7	2	1	0	2	13	4	2	4	3	5	2	5	4	2	2	3
94	C-domain of spore coat protein S	1PRS	91	-2.0	two	0.10	5	0	6	4	5	7	6	4	4	6	1	8	6	5	4	7	5	5	0	3
95	apocytochrome b5	1HKO	104	3.0	two	-0.96	5	0	7	14	3	6	5	8	9	8	0	3	3	2	3	10	9	4	1	4
96	ACBP	1ST7	86	8.5	multi	0.62	8	0	6	11	2	3	0	3	12	9	1	4	3	4	1	5	3	4	2	5
97	CAfn2	1K85	88	1.4	two	-0.71	18	0	4	0	1	6	0	2	3	4	0	5	2	1	0	10	20	8	1	3
98	N-domain of spore coat protein S	1PRS	88	3.0	two	0.07	8	0	4	5	3	7	0	7	3	6	0	10	6	5	2	5	3	11	0	3
99	hbLBD	1K8M	93	-0.7	two	-1.48	3	1	9	8	2	6	6	7	8	6	1	1	1	4	2	6	5	11	1	5
100	Third PDZ domain from PSD-95	1TP3	93	3.0	multi	0.11	8	0	4	8	4	13	2	10	3	7	0	4	3	4	7	6	3	5	0	2
101	PDZ2 domain from PTP-BL	1GM1	94	1.0	multi	-0.98	7	0	4	6	1	14	4	5	7	8	0	3	3	3	4	5	7	12	1	0
102	FRB	1AUE	95	6.0	multi	2.01	6	1	3	11	4	6	4	2	5	9	6	3	2	4	8	4	3	5	4	5
103	Death domain	1E41	100	6.9	two	1.77	9	3	8	8	1	4	1	4	5	10	1	7	1	6	11	5	4	9	2	1
104	P13	1QTU	117	-0.4	two	0.69	6	0	8	9	1	7	6	4	1	15	4	2	8	10	10	6	3	9	4	4
105	Myotrophin	2MYO	118	4.8	multi	1.61	12	3	11	8	2	10	4	4	12	18	2	4	4	3	2	3	5	7	1	3
106	Hisactophilin	1HCD	118	4.6	multi	-0.79	5	1	6	7	5	13	31	6	9	7	1	2	0	1	1	8	6	6	0	3
107	RNase-H	3H08	146	1.9	multi	1.67	15	0	6	10	1	12	3	7	14	14	5	10	5	2	7	9	9	6	5	6
108	Apoflavodoxin	3F6R	148	3.5	multi	3.30	23	2	10	18	8	14	2	7	6	15	4	5	2	4	3	10	4	8	1	2
109	Staphylococcal nuclease	1SNQ	149	2.3	multi	0.73	14	0	8	12	3	12	3	5	23	12	4	6	4	6	5	5	10	9	1	7
110	p19INK4d	1BI8	166	2.2	multi	1.46	21	0	12	9	4	15	8	3	3	25	4	3	7	8	12	7	9	16	0	0
111	Apoflavodoxin	1RCF	169	0.8	two	-1.57	8	1	20	11	8	19	1	11	10	15	0	8	1	9	4	11	10	10	4	8
112	sIGPS	1IGS	222	-7.8	multi	0.61	11	0	10	27	7	10	0	29	15	25	5	14	7	3	16	19	6	10	0	8
113	Carbonic anhydrase	5A25	260	-4.2	multi	-0.03	17	0	19	11	11	20	11	5	18	26	4	13	19	12	9	16	14	20	7	8
116	B1 domain of streptococcal protein G	3GB1	56	6.0	two	-0.31	6	0	5	5	2	4	0	1	6	3	1	3	0	1	0	0	11	4	1	3
